# Supplementary material for: Synchrotron-Based Fourier-Transform Infrared Micro-Spectroscopy of Cerebrospinal Fluid from Amyotrophic Lateral Sclerosis Patients Reveals a Unique Biomolecular Profile
Source: Cells. 2023 May 23;12(11):1451. doi: 10.3390/cells12111451 (PMC10253168; doi:10.3390/cells12111451)
Supplement: Supplementary file 1 [file cells-12-01451-s001.zip › cells-2312631-supplementary.pdf]

**Supplementary Table S1:** ALS patient data.

## ALS patients

| Pseudonym | Sex | ALS form | Age (years) | Disease duration (months) | ALSFRS-R | ALSFRS-R slope (pt./mo.) | pNfH (pg/ml) | Leukocyte count (cells/µl) | Total protein (mg/l) | Albumin quotient | lactate (mmol/l) | Genetics |
|---|---|---|---|---|---|---|---|---|---|---|---|---|
| M3 | f | bulbar | 73 | 9 | 37 | 1,2 | n.a. | 1 | 243 | 3,8 | 1,7 | |
| M5 | m | spinal | 56 | 3 | 45 | 1,0 | 2589 | 0 | 416 | 6,6 | 1,7 | |
| M7 | m | spinal | 49 | 11 | 45 | 0,3 | 2111 | 1 | 582 | 10,2 | 1,6 | C9orf72 |
| K9 | f | spinal | 65 | 4 | 45 | 0,8 | 1577 | 1 | 305 | 5 | 1,6 | |
| K12 | f | spinal | 53 | 8 | 44 | 0,5 | 1258 | 0 | 331 | 5,6 | 1,7 | |
| K13 | m | spinal | 53 | 15 | 44 | 0,3 | 4227 | 0 | 309 | 5,2 | 1,7 | |
| K14 | f | bulbar | 57 | 13 | 43 | 0,4 | 2319 | 1 | 363 | 5,7 | 1,5 | |
| K15 | m | spinal | 76 | 7 | 41 | 1,0 | 10518 | 1 | 445 | 8 | 1,5 | |
| K16 | f | spinal | 76 | 15 | 33 | 1,0 | 4065 | 2 | 656 | 12,6 | 1,6 | |
| K17 | f | spinal | 46 | 48 | 31 | 0,4 | 648 | 0 | 310 | 4,7 | 1,8 | |
| K19 | m | spinal | 80 | 6 | 35 | 2,2 | 7863 | 2 | 514 | 9,5 | 1,6 | |
| K25 | m | spinal | 76 | 7 | 39 | 1,3 | 1520 | 0 | 282 | 5,2 | 1,5 | |
| K26 | m | bulbar | 59 | 10 | 40 | 0,8 | 1725 | 0 | 615 | 10,4 | 1,8 | |
| K27 | m | spinal | 56 | 11 | 30 | 1,6 | 4000 | 1 | 458 | 7,6 | 1,8 | |
| K29 | f | spinal | 66 | 18 | 42 | 0,3 | 1614 | 1 | 605 | 9,9 | 1,4 | |
| G178 | f | spinal | 68 | 32 | 18 | 0,9 | 6786 | 5 | 949 | 17,9 | 3,6 | |
| Y1 | m | spinal | 61 | 18 | 34 | 0,8 | 4119 | 1 | 687 | 12,4 | 1,4 | |
| M10 | m | bulbar | 72 | 18 | 43 | 0,3 | 2181 | 0 | 591 | 11,3 | 1,4 | |
| M8 | m | spinal | 72 | 6 | 45 | 0,5 | 604 | 0 | 303 | 5,1 | 1,6 | |
| M9 | m | spinal | 63 | 72 | 47 | 0,0 | 244 | 6 | 403 | 6,8 | 1,6 | |
| M11 | f | spinal | 69 | 6 | 45 | 0,5 | 1229 | 1 | 279 | 3,6 | 1,7 | |
| M12 | m | bulbar | 69 | 6 | 44 | 0,7 | 3350 | 1 | 433 | 6,2 | 1,6 | |
| M13 | f | spinal | 55 | 10 | 45 | 0,3 | 3561 | 0 | 293 | 4,6 | 1,5 | |
| M14 | f | bulbar | 61 | 32 | 46 | 0,1 | 539 | 1 | 380 | 7,7 | 1,4 | |
| M15 | m | spinal | 55 | 6 | 36 | 2,0 | 5391 | 1 | 313 | 5,6 | 1,6 | |
| M16 | m | bulbar | 75 | 3 | 40 | 2,7 | 6345 | 0 | 443 | 8,7 | 1,6 | |
| M18 | m | spinal | 57 | 35 | 41 | 0,2 | 7057 | 0 | 527 | 9,4 | 1,7 | |
| M20 | f | bulbar | 68 | 30 | 34 | 0,5 | 4294 | 3 | 290 | 5,1 | 1,8 | C9orf72 |
| M21 | m | bulbar | 49 | 12 | 40 | 0,7 | 4176 | 0 | 395 | 7,4 | 1,8 | |
| M22 | m | spinal | 70 | 4 | 45 | 0,8 | 2991 | 2 | 410 | 7,3 | 1,5 | |
| M23 | m | spinal | 54 | 7 | 45 | 0,4 | 2298 | 1 | 390 | 6,8 | 1,5 | |
| M24 | m | spinal | 60 | 13 | 46 | 0,2 | 2187 | 0 | 316 | 4,2 | 1,8 | C9orf72 |
| M25 | m | spinal | 59 | 41 | 42 | 0,1 | 423 | 1 | 367 | 5,9 | 1,8 | |
| **Mean** | | | 63 | 16 | 40 | 0,7 | 3244 | 1 | 430 | 7 | 2 | |
| **Stand.-Dev.** | | | 9,2 | 15,3 | 6,2 | 0,6 | 2430,3 | 1,4 | 152,9 | 3,1 | 0,4 | |
| **Median** | | | 61 | 11 | 42 | 0,5 | 2454 | 1,0 | 395,0 | 6,8 | 1,6 | |
| **Minimum** | | | 46 | 3 | 18 | 0,0 | 244 | 0,0 | 243,0 | 3,6 | 1,4 | |
| **Maximum** | | | 80 | 72 | 47 | 2,7 | 10518 | 6,0 | 949,0 | 17,9 | 3,6 | |

## Controls

| Pseudonym | Sex | Diagnosis | Age (years) | Leukocyte count (cells/µl) | Total protein (mg/l) | Albumin quotient | lactate (mmol/l) |
|---|---|---|---|---|---|---|---|
| K1 | f | IIH | 49 | 2 | 520 | 9,4 | 1,4 |
| K2 | f | IIH | 57 | 2 | 498 | 8,9 | 1,5 |
| K4 | m | NPH | 88 | 0 | 386 | 5,9 | 2,1 |
| K6 | m | BFS | 56 | 0 | 338 | 5 | 1,5 |
| K8 | m | Ischemia | 78 | 1 | 556 | 9 | 1,5 |
| K10 | f | Dysesthesia | 38 | 1 | 323 | 4,8 | 1,4 |
| K11 | f | Dysesthesia | 26 | 0 | 176 | 2,3 | 1,6 |
| K22 | f | IIH | 46 | 5 | 305 | 5 | 1,4 |
| K32 | f | SMA | 22 | 2 | 420 | 6,5 | 1,2 |
| G237 | m | Cereb. Ataxia | 80 | 1 | 531 | 10,3 | 1,8 |
| G242 | m | Syncope | 80 | 1 | 318 | 5,1 | 1,8 |
| G243 | m | Syncope | 75 | 1 | 371 | 5,8 | 1,6 |
| G245 | f | Syncope | 54 | 1 | 450 | 7,6 | 1,6 |
| G248 | f | PNP | 77 | 0 | 349 | 5,9 | 1,2 |
| G249 | f | PNP | 64 | 0 | 393 | 8 | 1,4 |
| G243 | m | Syncope | 75 | 1 | 371 | 5,8 | 1,6 |
| X13 | m | ataxia | 47 | 0 | 438 | 6 | 1,3 |
| A80 | m | ET | 73 | 1 | 701 | 10 | 1,6 |
| G161 | m | dizziness | 59 | 2 | 440 | 5,6 | 1,5 |
| G197 | f | SKS | 71 | 0 | 1142 | 22,7 | 1,9 |
| G252 | m | CIDP | 68 | 2 | 456 | 6,5 | 2 |
| Q3 | f | Rheumatism | 52 | 0 | 427 | 8 | 1,3 |
| Q4 | f | SKS | 60 | 1 | 392 | 6,7 | 1,3 |
| P76 | m | Tremor | 61 | 2 | 531 | 9,2 | 1,5 |
| Q5 | m | /estibular failur | 76 | 1 | 439 | 7,3 | 1,6 |
| X17 | m | gait disorder | 77 | 1 | 713 | 11,5 | 1,6 |
| G160 | m | headache | 51 | 1 | 448 | 7,8 | 1,4 |
| G190 | f | PNP | 71 | 0 | 532 | 9 | 1,3 |
| B30 | m | MS | 43 | 1 | 373 | 5,2 | 1,4 |
| G231 | m | MS | 47 | 5 | 715 | 11,9 | 1,6 |
| G220 | m | PNP | 62 | 1 | 332 | 4,7 | 2,1 |
| G244 | m | TGA | 57 | 5 | 1140 | 22,4 | 1,7 |
| **Mean** | | | 61 | 1 | 485 | 8 | 2 |
| **Stand.-Dev.** | | | 16,0 | 1,4 | 208,9 | 4,4 | 0,2 |
| **Median** | | | 61 | 1 | 439 | 7 | 2 |
| **Minimum** | | | 22 | 0 | 176 | 2 | 1 |
| **Maximum** | | | 88 | 5 | 1142 | 23 | 2 |

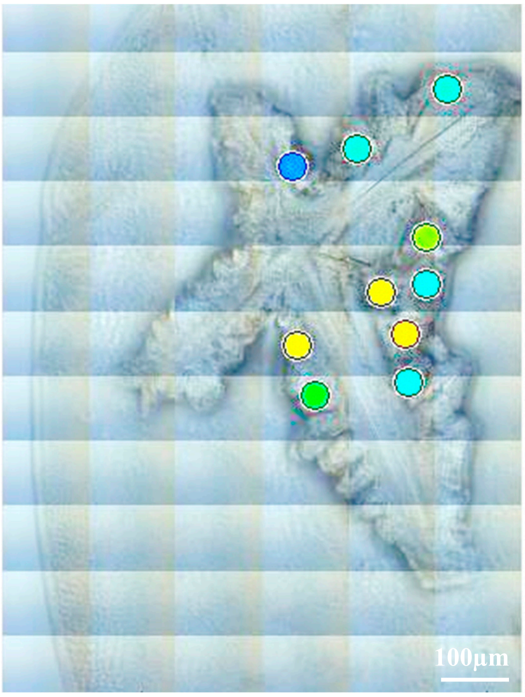


**Supplementary Figure S1:** Optical images of CSF sample after deposition on a $CaF_2$ IR window and water evaporation. The color points represent measured points with an aperture of 10 × 10 µm.

This figure represents an overview of the CSF samples prior to the FTIR measurements (composite picture). Six to ten different points per sample were taken for the IR acquisition and the average spectra were taken for further analysis.

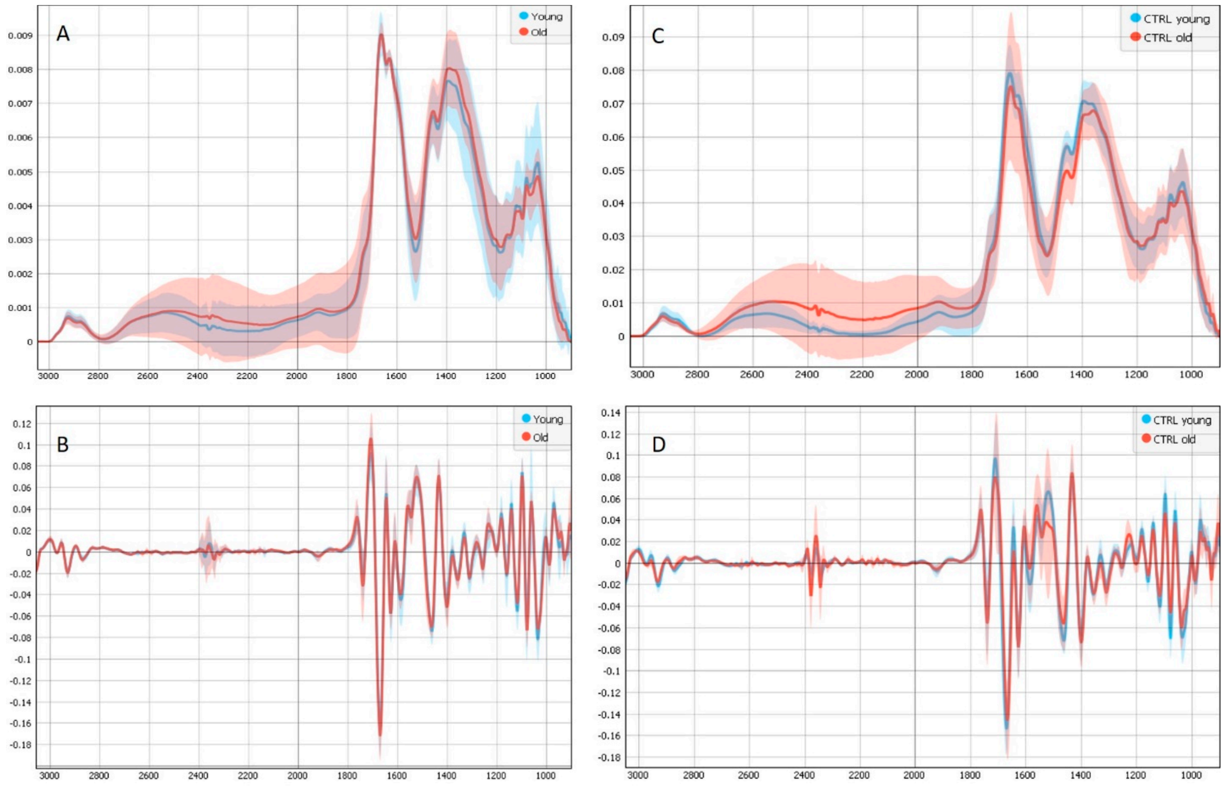


**Supplementary Figure S2**: **Young versus old ALS patients and controls.** Whole spectra (**A**) and the second derivate of the FTIR averaged spectra (**B**) of samples from young (under 65 years of age; blue) and old ALS patients (over 65 years of age; red) (N=18 and 15, respectively). Whole spectra (**C**) and the second derivate of the FTIR averaged spectra (**D**) of samples from young (blue) and old control patients (red) (N=13 and 16, respectively). Control groups (Figure S4C,D) showed more fluctuation of the samples in the fingerprint and lipids part of spectra. However, there are several similarities in the bands between control and ALS group, as for example bands at ~1036, 1120, 1465, 1585 $cm^{-1}$ more pronounced in the "young" samples, and assigned to nucleic acids, RNA, $CH_2$ and $CH_3$ deformation movements in proteins. In the group of "old" samples, the change at 930 $cm^{-1}$ were marked and this band at is ascribed to the Z-form of DNA. None of these changes was statistically significant.

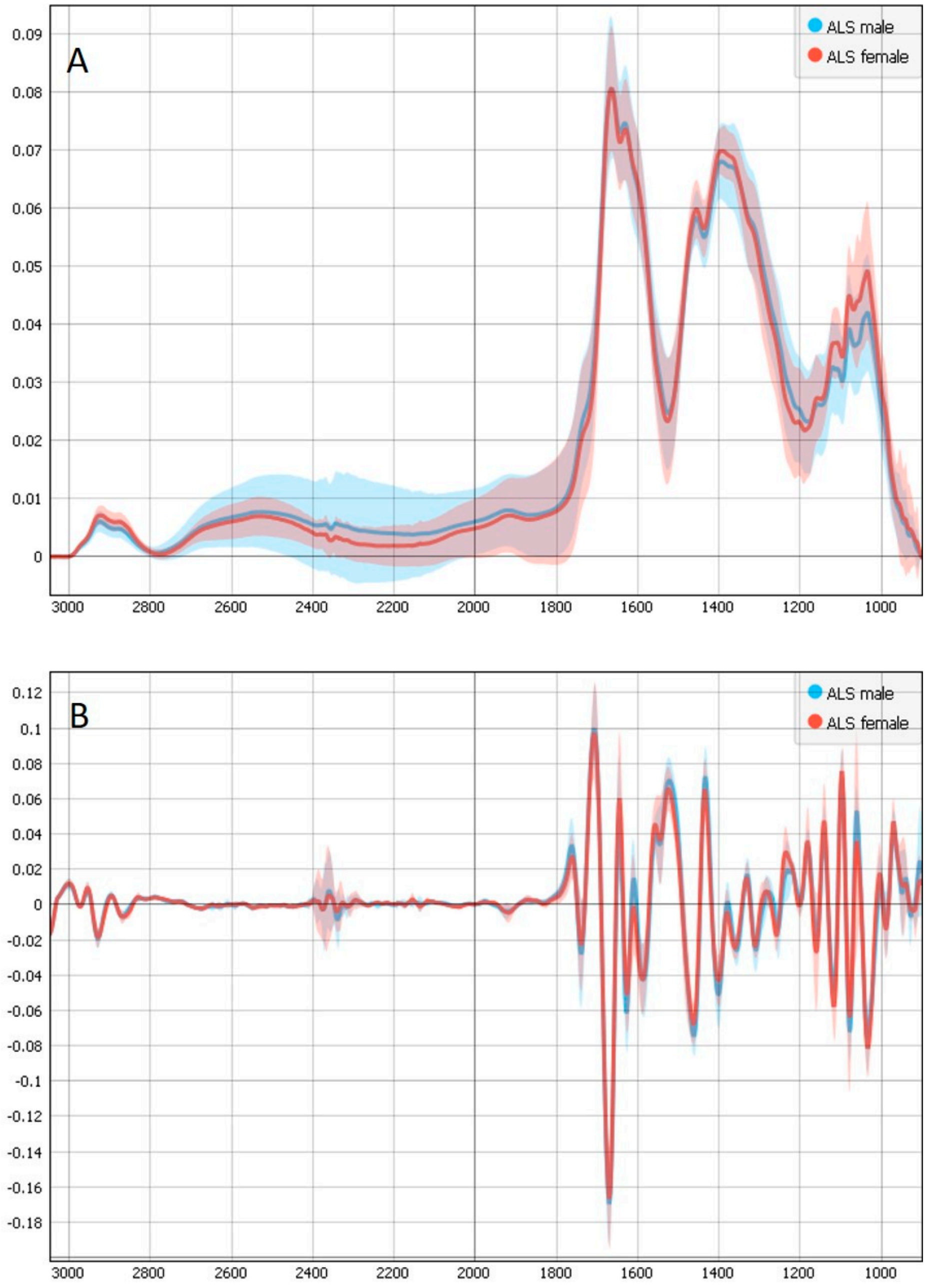


**Supplementary Figure S3: Male vs. female ALS patients**. Whole spectra (**A**) and the second derivate of the FTIR averaged spectra (**B**) of samples from male (blue) and female ALS patients (red) (N=21 and 12, respectively).

This figure presents the average spectra of the CSF samples from the ALS patients group divided into male and female subgroups. Comparison of spectra did not show significant differences. Nevertheless, there are small differences at 1077 and 1400 $cm^{-1}$, wavelength corresponding to carbohydrates and carboxyl group, which are more pronounced in the female samples. On the other hand, the male group had more pronounced bands at 1740, 1630, 1400, 1077 $cm^{-1}$ that correspond to carbonyl groups, β-sheet, carboxyl groups and glycogen groups.

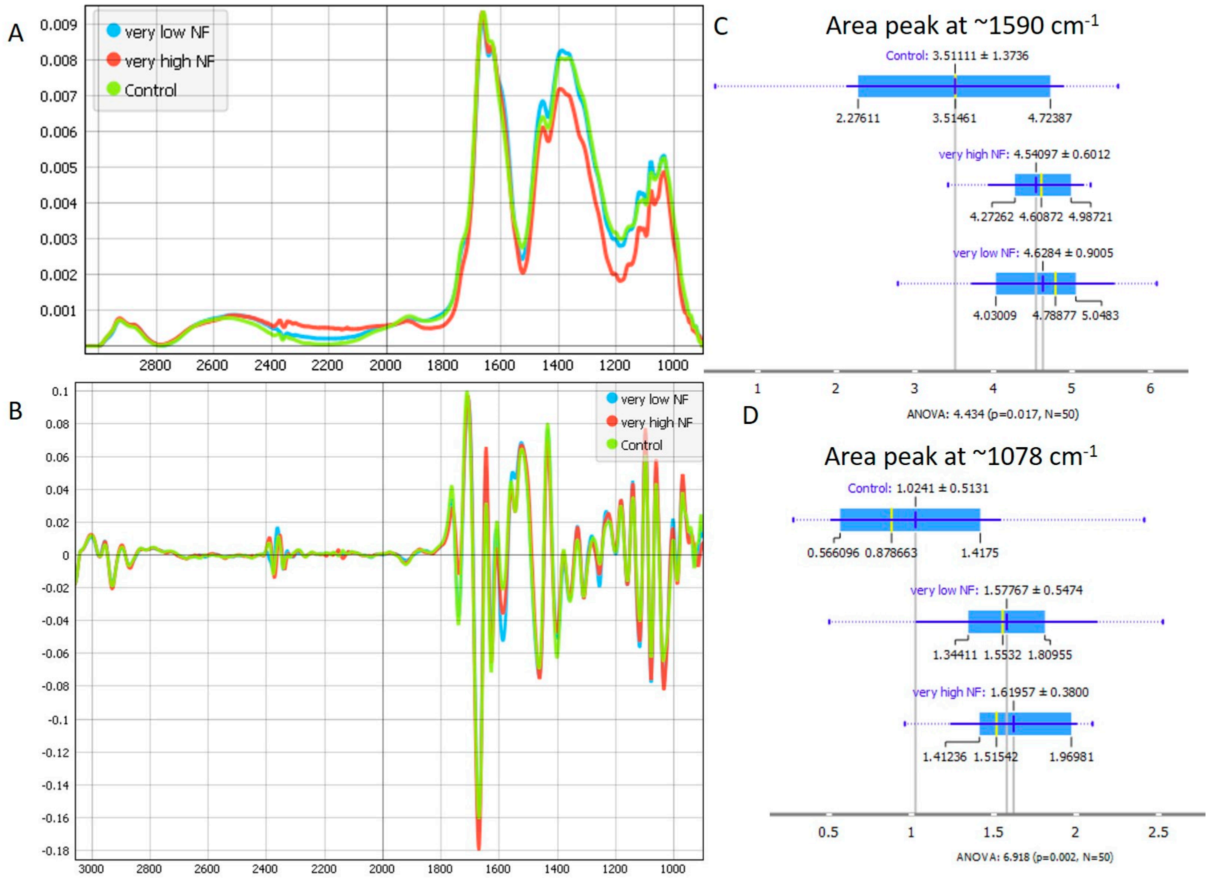


**Supplementary Figure S4: High vs. low neurofilament vs. control.** Whole spectra (**A**) and the second derivate of the FTIR averaged spectra (**B**) of samples from patients with relatively low levels of neurofilaments (pNfH <2000 pg/ml; blue) and from patients with relatively high levels of neurofilaments (pNfH >5000 pg/ml; red) and control samples (green). (**C**) Integrated area under peak at 1590 cm$^{-1}$. (**D**). Area under peak at 1078 cm$^{-1}$. (N=11 for low neurofilaments samples, 7 for high neurofilaments and 32 - control).

This figure presents the FTIR signature for relatively low (pNfH <2000 pg/ml; LNF) and relatively high (pNfH >5000 pg/ml; HNF) neurofilament levels in the CSF of ALS samples in comparison to control samples. Comparison of spectra show similar shape for control and LNF in comparison with HNF. Significant differences at 1078 cm$^{-1}$, which correspond to glycogen, and 1587 cm$^{-1}$ assigned to Glu and Asp and significant lower concentration was found in control samples for both macromolecules, as presented in panel 4C, D, respectively.

There are slight differences at 920, 1034 and 1160 cm$^{-1}$, wavelength corresponding to Z type of DNA, COH deformation movement in nucleic acids and C-O groups in in proteins and carbohydrates, which are more pronounced in the HNF group. Band at 1740 cm$^{-1}$, connected to ester groups, were similarly more abundant in the LNF and control in comparison to HNF.

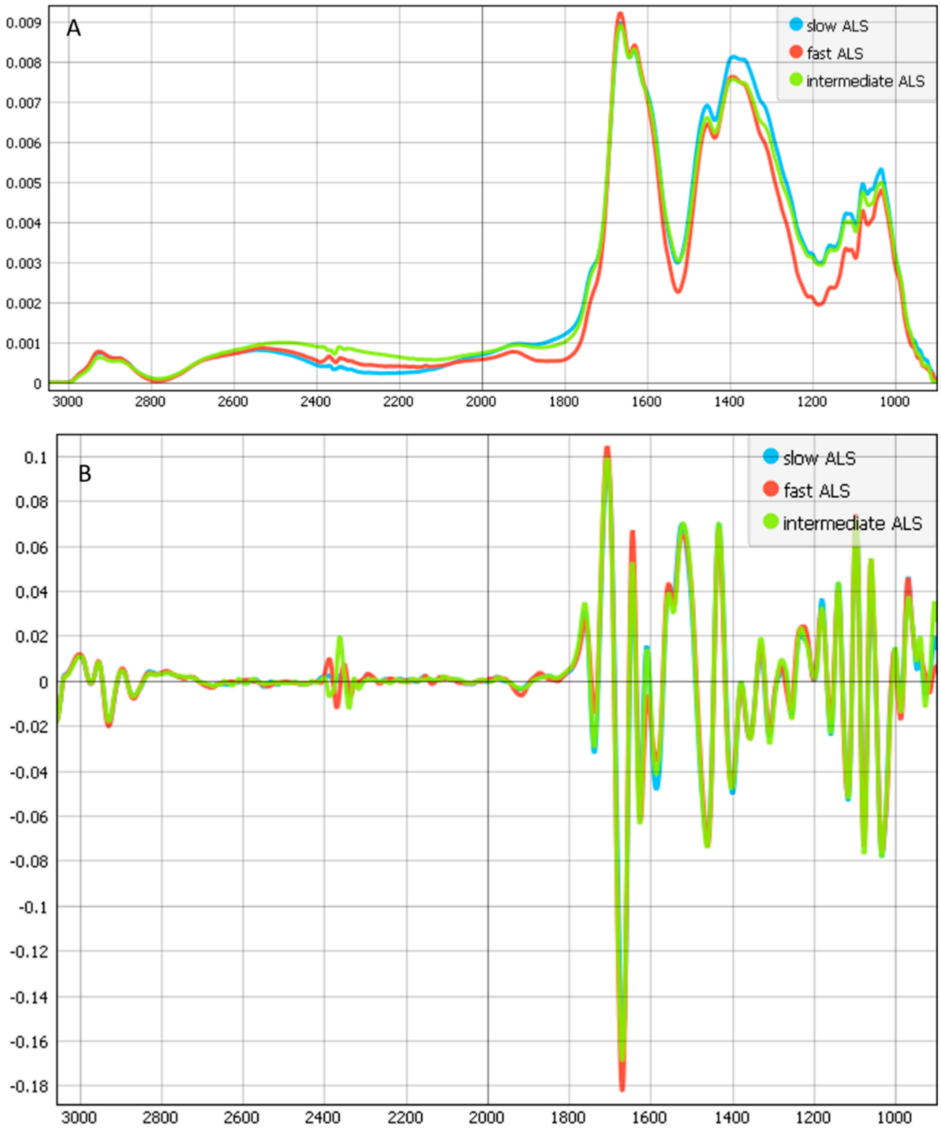


**Supplementary Figure S5: Fast vs. slow disease progression (ALSFRS-R slope).** Whole spectra (**A**) and the second derivate of the FTIR averaged spectra (**B**) of samples from ALS patients with a slow clinical progression (ALSFRS-R Slope <0.5 points per month; blue), intermediate progression (ALSFRS-R Slope between 0.5 and 1 points per month (incl. 0.5); green) and fast progression of disease (ALSFRS-R-Slope >1 point per month (incl. 1); red) (N=9, 10 and 14, respectively).

Figure S6 shows FTIR spectroscopy of ALS samples with fast progression, intermediate and fast progression based on the ALSFRS-R-slope. There are differences observed mostly for fast progression of disease at ~920, 1079, 1107, 1627, and 1670 $cm^{-1}$, wavelength corresponding to Z-DNA movements, glycogen, RNA conformational changes, and of fibrillary β-sheet and β-turn and loops (proteins conformational changes). On the other hand, the slow ALS progression displays more pronounced bands at 1160 $cm^{-1}$, 1585 and 1740 $cm^{-1}$ which are connected to glycogen, amino-acids chains as Glu and/or Asp, respectively. However, none of these changes was statistically significant.

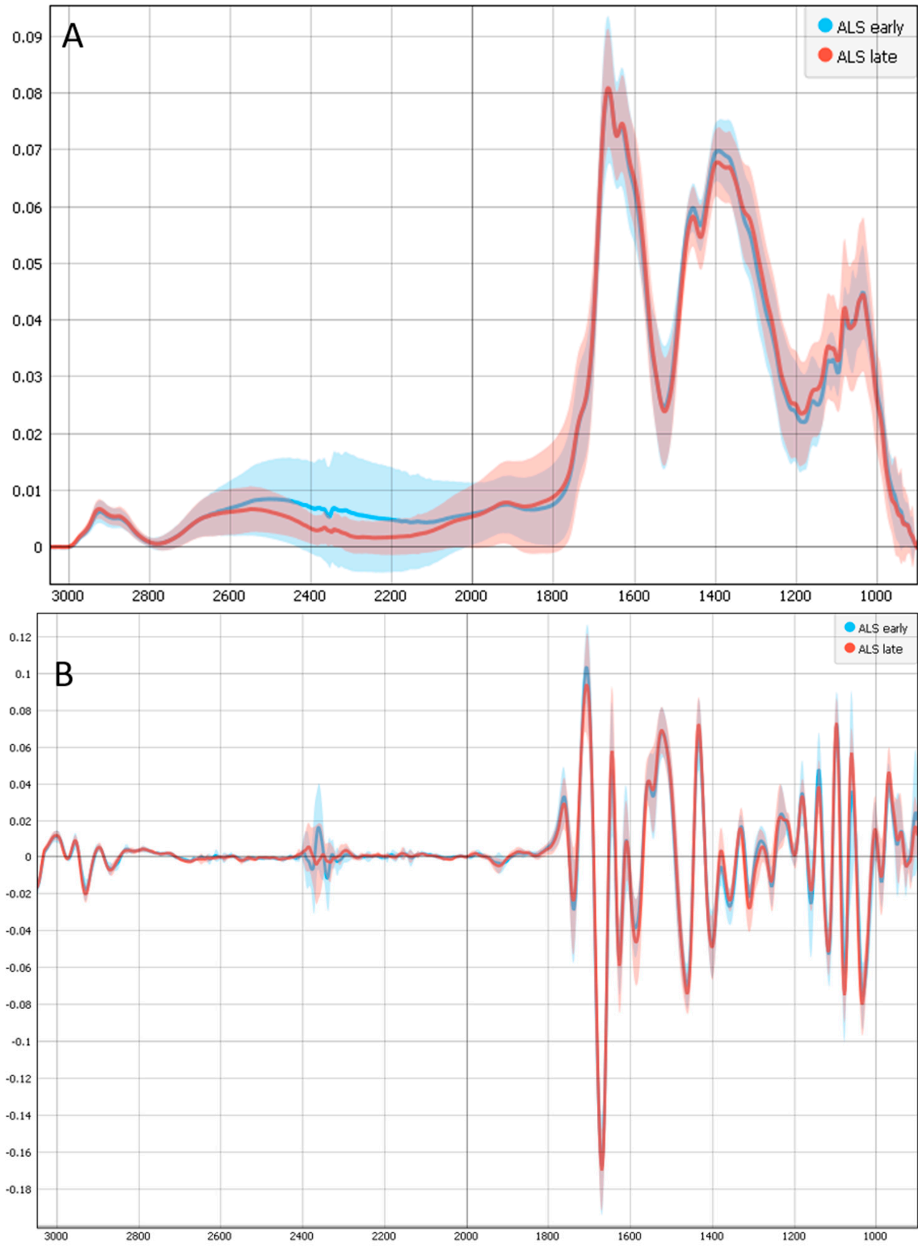


**Supplementary Figure S6: Early versus late disease stage.** Whole spectra (**A**) and the second derivate of the FTIR averaged spectra (**B**) of samples from early stage (<11 months after symptom onset) (blue) and late stage (>11months after symptom onset) (red) ALS patients (N=17 and 16, respectively).

This figure presents the signature of the FTIR for early stage (<11 months of disease duration) and late stage (>11 months of disease duration) of the ALS patients. Comparison of spectra did not show significant differences. However, there are slight differences possible to read at 1160, 1360, and 1740 cm$^{-1}$, wavelength corresponding to and C-O groups in in proteins and carbohydrates, $CH_2$ and/or $CH_3$ vibration and carbonyl groups, which are more pronounced in early stage of disease. From the other side, the in the late stage of ALS, there are more pronounced band at 1310 cm$^{-1}$, which correspond to Amide III, and 1587 cm$^{-1}$ assigned to Glu and/or Asp.

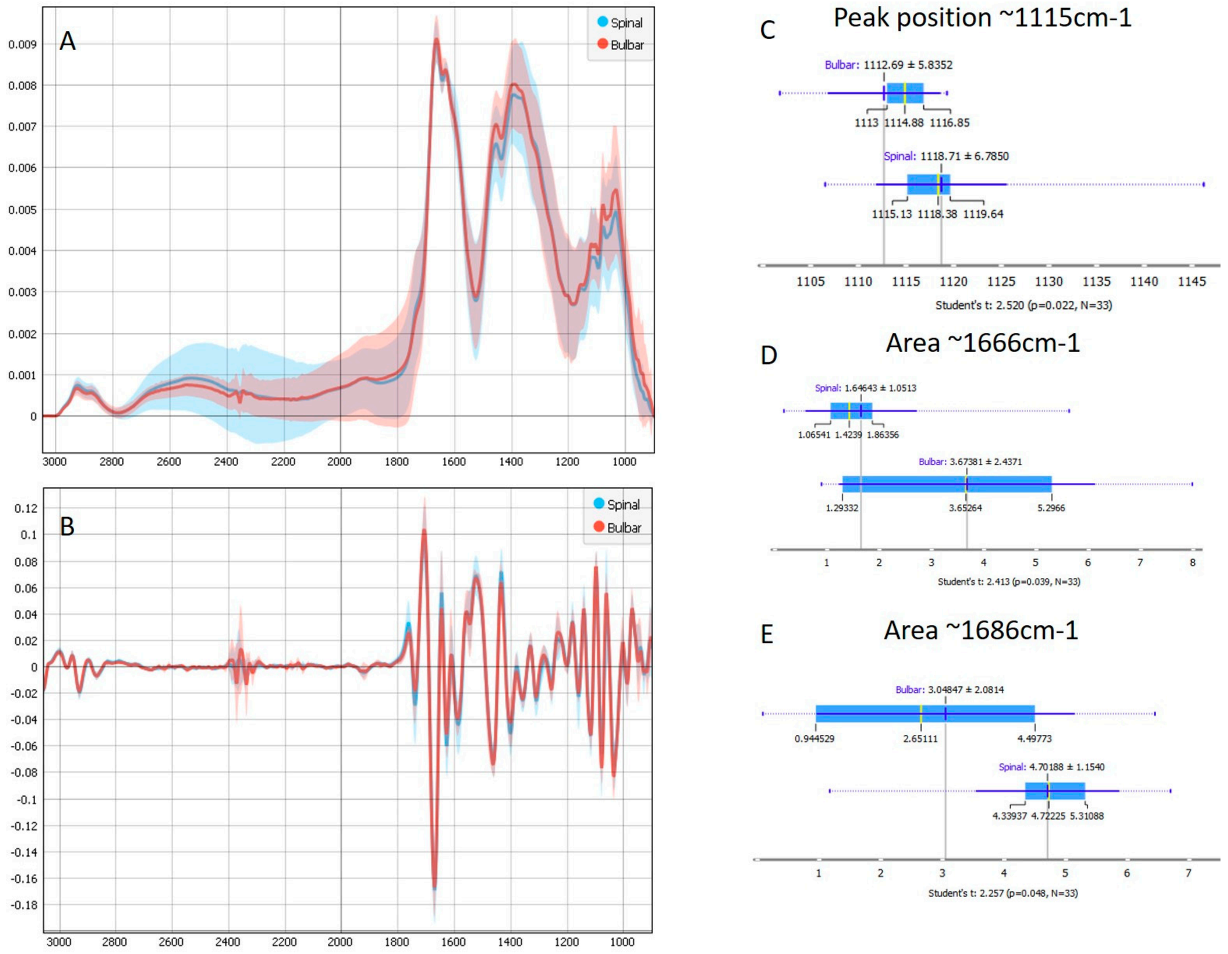


**Supplementary Figure S7: Spinal vs. bulbar ALS.** Whole spectra (**A**) and the second derivate of the FTIR averaged spectra (**B**) of spinal (blue) and bulbar ALS samples (red) (N=24 and 9, respectively). (**C**) is showing the significant change in the peak position at ~1115 $cm^{-1}$. (**D**): Area under the $\alpha$-helix structure ~1666 $cm^{-1}$ E: Area under the β-turn and loops structure ~1686 $cm^{-1}$.

This figure presents the FTIR spectra of the CSF samples from ALS patients with spinal versus bulbar subtype. The comparison of spectra shows a shift of the position of RNA from 1113 $cm^{-1}$ for the spinal patients to 1119 $cm^{-1}$ for the bulbar patients. Regarding the protein structure, there is a significant difference regarding the $\alpha$-helix band (~1664 $cm^{-1}$), which is significantly higher in bulbar samples, while the β-turn and loops band (~1680 $cm^{-1}$) is significantly more pronounced in spinal ALS samples.

Besides, there are slight differences at the bands of ~ 920, 1034, 1078 and 1120 $cm^{-1}$, more noticeable in bulbar samples, while in the spinal samples peaks at 1740, 1630, 1590 and 1400 $cm^{-1}$ are more prominent. These changes corresponding to Z type of DNA, nucleic acids, carbohydrates, and RNA which are more pronounced in bulbar ALS samples. On the other hand, the spinal samples had more evident bands of carbonyl groups, β-sheet, amino acids Glu and/or Asp and carboxyl groups.
